# Sumoylation Protects Against β-Synuclein Toxicity in Yeast

**DOI:** 10.3389/fnmol.2018.00094

**Published:** 2018-03-27

**Authors:** Blagovesta Popova, Alexandra Kleinknecht, Patricia Arendarski, Jasmin Mischke, Dan Wang, Gerhard H. Braus

**Affiliations:** ^1^Department of Molecular Microbiology and Genetics and Göttingen Center for Molecular Biosciences (GZMB), Institute for Microbiology and Genetics, Universität Göttingen, Göttingen, Germany; ^2^Center for Nanoscale Microscopy and Molecular Physiology of the Brain (CNMPB), Göttingen, Germany

**Keywords:** beta-synuclein, Parkinson’s disease, sumoylation, posttranslational modification, yeast, proteasome, autophagy, aggregation

## Abstract

Aggregation of α-synuclein (αSyn) plays a central role in the pathogenesis of Parkinson’s disease (PD). The budding yeast *Saccharomyces cerevisiae* serves as reference cell to study the interplay between αSyn misfolding, cytotoxicity and post-translational modifications (PTMs). The synuclein family includes α, β and γ isoforms. β-synuclein (βSyn) and αSyn are found at presynaptic terminals and both proteins are presumably involved in disease pathogenesis. Similar to αSyn, expression of βSyn leads to growth deficiency and formation of intracellular aggregates in yeast. Co-expression of αSyn and βSyn exacerbates the cytotoxicity. This suggests an important role of βSyn homeostasis in PD pathology. We show here that the small ubiquitin-like modifier SUMO is an important determinant of protein stability and βSyn-induced toxicity in eukaryotic cells. Downregulation of sumoylation in a yeast strain, defective for the SUMO-encoding gene resulted in reduced yeast growth, whereas upregulation of sumoylation rescued growth of yeast cell expressing βSyn. This corroborates a protective role of the cellular sumoylation machinery against βSyn-induced toxicity. Upregulation of sumoylation significantly reduced βSyn aggregate formation. This is an indirect molecular process, which is not directly linked to βSyn sumoylation because amino acid substitutions in the lysine residues required for βSyn sumoylation decreased aggregation without changing yeast cellular toxicity. αSyn aggregates are more predominantly degraded by the autophagy/vacuole than by the 26S ubiquitin proteasome system. We demonstrate a vice versa situation for βSyn, which is mainly degraded in the 26S proteasome. Downregulation of sumoylation significantly compromised the clearance of βSyn by the 26S proteasome and increased protein stability. This effect is specific, because depletion of functional SUMO did neither affect βSyn aggregate formation nor its degradation by the autophagy/vacuolar pathway. Our data support that cellular βSyn toxicity and aggregation do not correlate in their cellular impact as for αSyn but rather represent two distinct independent molecular functions and molecular mechanisms. These insights into the relationship between βSyn-induced toxicity, aggregate formation and degradation demonstrate a significant distinction between the impact of αSyn compared to βSyn on eukaryotic cells.

## Introduction

Parkinson’s disease (PD) is characterized by loss of dopaminergic neurons in the *substantia nigra* in the midbrain and formation of intracellular protein inclusions called Lewy bodies (Kalia and Lang, 2015). Loss of dopaminergic neurons leads to dopamine depletion and results in a wide range of motoric and non-motoric symptoms. One of the major components of Lewy bodies is the small and highly abundant protein α-synuclein (αSyn; Spillantini et al., 1998). αSyn is part of a family of proteins that includes also β-synuclein (βSyn) and γ-synuclein (γSyn). αSyn and βSyn are localized predominantly at presynaptic nerve terminals, whereas γSyn is abundant in the peripheral nervous system (Li et al., 2002; Mori et al., 2002). Synuclein family members share a highly conserved N-terminal domain and a more divergent and highly acidic C-terminal domain. αSyn and βSyn are closely related to each other and their sequences share 62% homology (George, 2002). The most notable difference between the two proteins is that βSyn lacks 11 residues in the central most hydrophobic region, referred to as non-amyloid-β component (NAC) region, involved in fibril formation and aggregation. All three synucleins are intrinsically unstructured when isolated under physiological conditions and adopt helical structures in their N-terminal domains upon binding to lipid vesicles (Sung and Eliezer, 2006; Ducas and Rhoades, 2012).

αSyn is implicated as prime contributor of PD development as it is associated with familial and sporadic forms of PD. The protein has a strong propensity to self-assemble into oligomeric protofibrils that can further mature into different types of fibrils and insoluble aggregates (Villar-Piqué et al., 2015). The abnormal accumulation of the protein is partly triggered by gene duplications or even triplications as well as missense mutations (Polymeropoulos et al., 1997; Krüger et al., 1998; Singleton et al., 2003; Zarranz et al., 2004). Aggregation of αSyn is assumed to constitute the central pathological process in synucleinopathies. One of the critical factors that affect αSyn aggregation process is the protein level in the cell, which depends on the rate of its turnover. αSyn can be degraded both by ubiquitin-proteasome system (UPS) and the autophagy/lysosome pathway (Webb et al., 2003; Xilouri et al., 2013). Inhibition of protein degradation pathways resulting in inefficient protein clearance leads to accumulation of pathological αSyn species and is sufficient to trigger neurotoxicity (Xilouri et al., 2013; Vilchez et al., 2014). Therefore, understanding of αSyn turnover mechanism is an essential aspect to uncover the pathological mechanism of PD.

αSyn undergoes numerous post-translational modifications (PTMs) such as phosphorylation, ubiquitination, sumoylation, nitration or acetylation (Giasson et al., 2000; Shimura et al., 2001; Fujiwara et al., 2002; Hasegawa et al., 2002; Dorval and Fraser, 2006; Kleinknecht et al., 2016; de Oliveira et al., 2017). PTMs influence αSyn aggregation and toxicity and in addition modulate the degradation of the protein by different proteolytic pathways (Popova et al., 2015). The small ubiquitin-like modifier SUMO is covalently conjugated to target proteins and modulates a number of cellular processes. Many PD-associated proteins are SUMO-modified, highlighting the importance of this PTM in neurodegeneration (Eckermann, 2013). αSyn is a substrate of SUMO1, one of the four SUMO isoforms in human (Dorval and Fraser, 2006). Sumoylation of αSyn negatively regulates αSyn aggregation by promoting protein solubility (Krumova et al., 2011).

The yeast *Saccharomyces cerevisiae* is a valuable model system for studying protein misfolding and cellular pathways associated with protein quality control systems due to the high conservation of functions with higher eukaryotes including humans (Menezes et al., 2015). Heterologous expression of αSyn in yeast results in aggregate formation and dose-dependent cytotoxicity, recapitulating central features of PD (Outeiro and Lindquist, 2003; Petroi et al., 2012). Yeast was employed to investigate the role of PTMs for αSyn aggregate clearance. Recent study demonstrated that αSyn is sumoylated in yeast and that the cellular mechanism of sumoylation is conserved from yeast to human (Shahpasandzadeh et al., 2014). The distribution of αSyn to different cellular degradation pathways was dependent on a complex cross-talk between sumoylation and phosphorylation at Serine 129 (S129).

The other two members of synuclein family have been less well studied until recently due to lack of genetic links to PD. Initial reports suggested βSyn as a negative regulator of αSyn aggregation (Hashimoto et al., 2001; Uversky et al., 2002; Fan et al., 2006). Recent results revealed that βSyn can act as a neurodegeneration-inducing factor and can cause cell loss when expressed in rat brains (Taschenberger et al., 2013). This suggests a role of βSyn in the pathogenesis of PD. V70M or P123H mutations in βSyn were linked to cases of dementia with Lewy bodies (DLB; Ohtake et al., 2004) and were suggested to be involved in lysosomal pathology (Wei et al., 2007). Expression of P123H βSyn in transgenic mouse resulted in progressive neurodegeneration that acted synergistically with overexpression of αSyn (Fujita et al., 2010). These studies revealed an emerging role of βSyn in a broad range of α-synucleinopathies. However, the molecular mechanism of this role remains poorly studied.

Recently, yeast was used as a reference cell to investigate the cellular effects of βSyn. Expression of βSyn but not γSyn was toxic to yeast cells and resulted in formation of cytosolic inclusions, similar to those formed by αSyn (Tenreiro et al., 2016). βSyn can induce cell death by mechanisms associated with vesicular trafficking impairment or oxidative stress, similarly as it is described for αSyn toxicity. Co-expression of αSyn and βSyn enhanced the cytotoxicity due to increased dosage of toxic species in an additive manner. Both different synucleins were able to form heterodimers in yeast as well as in human cells. These findings emphasize pathogenesis-related cellular role of βSyn, suggesting that increased levels of βSyn enhance αSyn-induced cytotoxicity due to dosage effects. The molecular mechanisms that control βSyn turnover in the cell are yet elusive.

In contrast to αSyn, PTM of βSyn are hardly studied. βSyn has been found to be phosphorylated at S118 *in vitro* and *in vivo* (Nakajo et al., 1993; Mbefo et al., 2010), however other PTMs were not yet reported. Since αSyn and βSyn proteins are highly similar, there have to be differences in their molecular behavior that might explain their differential contribution to neurodegeneration.

We used yeast as a reference cell to exploit the molecular mechanisms that affect βSyn turnover in the cell. We showed that SUMO is an important modulator of protein stability. βSyn is sumoylated in yeast at three lysine residues and sumoylation supports aggregate formation. The exchange of the codons for the sumoylation sites decreased βSyn aggregate formation but did not change cellular cytotoxicity monitored as yeast growth. However, downregulation of the entire cellular sumoylation pool drastically enhanced βSyn-induced cytotoxicity. We demonstrate in yeast cells that intact sumoylation machinery is prerequisite for the degradation of soluble as well as aggregated βSyn protein. βSyn is degraded mainly by the 26S proteasome, in contrast to αSyn that is degraded predominantly by the autophagy/vacuolar pathway. These findings reveal distinct molecular mechanisms of protein turnover for βSyn in comparison to αSyn.

## Materials and Methods

### Plasmid Construction, Yeast Strains, Transformation and Growth Conditions

Plasmids and *Saccharomyces cerevisiae* strains are listed in Tables 1, 2. Yeast plasmids expressing *SNCB-GFP* fusion were cloned into the *Sma*I site of the integrative plasmids pRS304 or pRS306 using GENEART Seamless cloning and assembly kit (Life technologies). The βSyn mutant constructs were generated by site-directed mutagenesis of the corresponding plasmids. All constructs were verified by DNA sequencing.

**Table 1:** Plasmids used in this study.

Name	Description	Source
p426	*URA3*, 2 μm, pUC origin, AmpR	Mumberg et al. (1994)
pRS304	*TRP1*, pUC origin, AmpR	Sikorski and Hieter (1989)
pRS306	*URA3*, pUC origin, AmpR	Sikorski and Hieter (1989)
pME3759	p426-*GAL1-GFP*	Petroi et al. (2012)
pME3763	p426-*GAL1::SNCA::GFP*	Petroi et al. (2012)
pME4102	p426-*GAL1::SNCB::GFP*	Tenreiro et al. (2016)
pME4103	p426-*GAL1::SNCG::GFP*	Tenreiro et al. (2016)
pME4624	p426-*GAL1::SNCB^K12R^::GFP*	This study
pME4625	p426-*GAL1::SNCB^K85R^::GFP*	This study
pME4626	p426-*GAL1::SNCB^K94R^::GFP*	This study
pME4627	p426-*GAL1::SNCB^K85R K94R^::GFP*	This study
pME4628	p426-*GAL1::SNCB^K12 K85R K94R^::GFP*	This study
pME4629	pRS304-*GAL1::SNCB*	This study
pME4630	pRS304-*GAL1::SNCB^K12R^*	This study
pME4631	pRS304-*GAL1::SNCB^K85R^*	This study
pME4632	pRS304-*GAL1::SNCB^K94R^*	This study
pME4633	pRS304-*GAL1*::*SNCB^K85R K94R^*	This study
pME4634	pRS304-*GAL1*::SNCB*^K12R K85R K94R^*	This study
pME4635	pRS306-*GAL1::SNCB::GFP*	This study

**Table 2:** Yeast strains used in this study.

Name	Genotype	Source
W303-1A	*MATa; ura3-1; trp1-1; leu2-3_112; his3-11; ade2-1; can1-100*	EUROSCARF
RH3465	W303 containing *GAL1::GFP* in *ura3* locus	Petroi et al. (2012)
RH3468	W303 containing three genomic copies of *GAL1::SNCA^WT^::GFP* in *ura3* locus	Petroi et al. (2012)
*ulp1^ts^*	ulp1ts-333	Li and Hochstrasser (1999)
RH3603	*ulp1^ts^* containing YIplac211-*ADH*-*His6*-*SMT3* in *his3* locus	Shahpasandzadeh et al. (2014)
*smt3^ts^*	S542:* MAT* α*, smt3-331*	Dieckhoff et al. (2004)
RH3700	W303 containing one genomic copy of *GAL1::SNCB^WT^::GFP* in *ura3* locus	This study
RH3701	W303 containing two genomic copies of *GAL1::SNCB^WT^::GFP* in *ura3* locus	This study
RH3702	W303 containing three genomic copies of *GAL1::SNCB^WT^::GFP* in *ura3* locus	This study
RH3703	RH3603 containing *GAL1::SNCB^WT^* in *trp1* locus	This study
RH3704	RH3603 containing *GAL1::SNCB^K12R^* in *trp1* locus	This study
RH3705	RH3603 containing *GAL1::SNCB^K85R^* in *trp1* locus	This study
RH3706	RH3603 containing *GAL1::SNCB^K94R^* in *trp1* locus	This study
RH3707	RH3603 containing *GAL1::SNCB^K85R K94R^* in *trp1* locus	This study
RH3708	RH3603 containing *GAL1::SNCB^K12R K85R K94R^* in *trp1* locus	This study
RH 3709	*smt3^ts^* containing one genomic copy of *GAL1::SNCB^WT^*::GFP in *ura3* locus	This study
RH 3710	*smt3^ts^* containing one genomic copy of *GAL1::SNCB^K12R K85R K94R^*::GFP in *ura3* locus	This study

The *GAL1-SNCB*-GFP was integrated into the mutated *ura3-1* locus of *S. cerevisiae* W303-1A strain using an intact *URA3* gene on the corresponding integrative plasmid for selection. The number of the integrated copies was determined by Southern hybridization as described previously (Petroi et al., 2012). The *GAL1::SNCB* or its derivatives were integrated into the *trp1-1* locus.

*S. cerevisiae* strains W303-1A, *smt3^ts^* and *ulp1^ts^* were used for transformations performed by standard lithium acetate protocol (Gietz et al., 1992). All strains were grown in Synthetic complete medium (SC; Guthrie and Fink, 1991) lacking the nutrient amino acid corresponding to the marker, and supplemented with 2% raffinose or 2% glucose. αSyn or βSyn expression was induced by shifting yeast cells cultivated overnight in raffinose to 2% galactose-containing medium (OD_600_ = 0.1).

### Spotting Assay

For growth test on solid medium, yeast cells were pre-grown in minimal medium containing 2% raffinose lacking the corresponding marker to mid-log phase. Cells were normalized to equal densities, serially diluted 10-fold starting with an OD_600_ of 0.1, and spotted on SC-plates containing either 2% glucose or 2% galactose and lacking in corresponding marker. *smt3^ts^* mutant cells were incubated at permissive temperature (25°C) and restrictive temperature (30°C). After 3 days incubation the plates were photographed.

### Growth Analysis in Liquid Culture

For growth tests in liquid cultures, cells were pre-grown in 2% raffinose-containing selective SC medium until logarithmic growth phase and inoculated in 2% galactose-containing SC medium to equal densities of OD_600_ = 0.1. Optical density measurements of 150 μl cell cultures were performed in quadruplicates in 96-well plates for 24 h using a microplate reader with temperature control and continuous shaking (Infinite M200, Tecan).

### Fluorescence Microscopy and Quantifications

Wild type (W303-1A) yeast cells harboring βSyn were grown in SC selective medium containing 2% raffinose at 30°C and *smt3^ts^* mutant cells at 25°C overnight, and transferred to 2% galactose containing medium for induction of βSyn expression for 6 h. *smt3^ts^* mutant cells were induced at 25°C or 30°C. Fluorescent images were obtained with Zeiss Observer. Z1 microscope (Zeiss) equipped with a CSU-X1 A1 confocal scanner unit (YOKOGAWA), QuantEM:512SC digital camera (Photometrics) and SlideBook 6.0 software package (Intelligent Imaging Innovations). For quantification of aggregation at least 200 cells were counted per strain and per experiment. The number of cells presenting inclusions was referred to the total number of cells counted. The values are mean of at least three independent experiments.

### Immunoblotting

Wild type (W303-1A) yeast cells harboring βSyn were pre-grown at 30°C in SC selective medium containing 2% raffinose. Cells were transferred to SC medium containing 2% galactose at OD_600_ = 0.1 to induce the *GAL1* promoter for 6 h. *smt3^ts^* or *ulp1^ts^* cells harboring βSyn were pre-incubated at 25°C and later transferred to either 25°C or 30°C. Total protein extracts were prepared and the protein concentrations were determined with a Bradford assay. Forty microgram of each protein were subjected to 12% SDS-polyacrylamide gel electrophoresis and transferred to a nitrocellulose membrane. Membranes were probed with βSyn rabbit monoclonal antibody (abcam, UK), GFP rat monoclonal antibody (Chromotek, Germany), SUMO rabbit polyclonal antibody (Rockland Immunochemicals Inc., USA) or ubiquitin mouse monoclonal antibody for detection of poly-ubiquitinated and ubiquitinated proteins (clone P4D1-A11, Millipore). GAPDH mouse monoclonal antibody (Thermo Fisher Scientific, USA) was used as loading controls. Pixel density values for Western quantification were obtained from TIFF files generated from digitized X-ray films (KODAK) and analyzed with the ImageJ software (NIH, Bethesda, MD, USA). Before comparison, sample density values were normalized to the corresponding loading control.

### Ultracentrifugation and Fractionation

The sedimentation assay, extraction of SDS-soluble and insoluble βSyn protein fractions were performed as described (Alberti et al., 2010) with modifications. Equal number of yeast cells corresponding to total OD = 10 were collected by centrifugation and resuspended in lysis buffer (50 mM Tris-HCl pH 7.5, 1 mM EDTA, 5 mM DTT, 1× protease inhibitor mix (Roche). The cells were lysed by shaking with glass beads at 4°C. The crude lysate was centrifuged for 5 min at 500 *g* to pellet the cell debris. Two-hundred microliter of each cleared lysate was centrifuged at 100,000 *g* for 30 min. The supernatant was designated as soluble protein. The pellet was washed three times with the lysis buffer, resuspended in 200 μl lysis buffer containing 2% SDS and incubated on ice for 30 min. The suspension was centrifuged for 30 min at 100,000 *g* and the supernatant was labeled as SDS-soluble protein fraction. The pellet was washed three times with lysis buffer, resuspended in 200 μl 6 M urea and designated as SDS-insoluble fraction. Equal amount from each fraction (20 μl) was analyzed by SDS-PAGE and Western blotting.

### Flow Cytometry

Cells were grown as above and protein expression was induced as described for 6 h. Before measuring, cells were re-suspended in 50 mM trisodium citrate buffer, pH 7.0. Flow cytometry analysis was performed on a BD FACSCANTO II (Becton Dickinson). Fifty thousand events were counted for each experiment. Data analysis was performed using the BD FACSDIVA software (Becton Dickinson). Representative examples that are shown in the Figures were repeated at least three times. Yeast cell membrane integrity was analyzed with PI staining. Yeast cells were incubated with 12.5 μg/ml PI for 30 min. As a positive control, cells were boiled for 10 min at 95°C.

### Promoter Shut-Off Assay and Chemical Treatments

Yeast cells carrying βSyn were pre-grown in selective SC medium containing 2% raffinose overnight and shifted to 2% galactose-containing selective SC medium to induce the βSyn expression for 4 h. Afterwards, cells were shifted to SC medium supplemented with 2% glucose to shut-off the promoter. For experiments with temperature sensitive yeast strain *smt3^ts^*, pre-incubation was performed at 25°C. Induction of βSyn expression and the promoter shut-off assay were performed at 25°C and 30°C. Five hours after promoter shut-off, cells were visualized by fluorescence microscopy. The reduction of number of cells displaying βSyn inclusions was recorded. To study the lysosome/vacuole degradation pathway (autophagy) phenylmethanesulfonyl fluoride (PMSF) in ethanol (EtOH) was applied to the suspension in a final concentration of 1 mM. For impairment of the proteasomal degradation system Carbobenzoxyl-leucinylleucinyl-leucinal (MG132) dissolved in dimethyl sulfoxide (DMSO) was added to the cell suspension in a final concentration of 75 μM. Drug treatment was conducted concomitantly with the shift to glucose-supplemented medium. In parallel, equal volume of DMSO or EtOH was applied to the control cells. For drug treatment with MG132 induction-medium containing galactose and shut-off-medium containing glucose was supplemented with 0.003% SDS and 0.1% proline.

### Ni^2+^-NTA Affinity Chromatography

*ulp1^ts^* mutant cells carrying *GAL1-SNCB* integrations and His6-tagged Smt3 (*His6-SMT3*) were pre-grown in 200 ml SC medium containing 2% raffinose at 30°C overnight. Total cells harvested by centrifugation were transferred to 2 l YEPD liquid medium containing 2% galactose for 12 h induction. Cells were collected and lysed by 25 ml 1.85 M NaOH containing 7.5% ß-mercaptoethanol for 10 min on ice. Proteins were precipitated in 25 ml 50% trichloroacetic acid (TCA), washed with 100% cold acetone, and suspended in 25 ml buffer A (6 M guanidine HCl, 100 mM sodium phosphate, 10 mM Tris/HCl, pH 8.0) and rotated for 1 h at 25°C. The supernatant was cleared by centrifugation; the pH was adjusted to 7.0 by 1 M Tris base and supplemented with imidazole to final concentration of 20 mM. After equilibration of the His GraviTrap column (GE Healthcare Life Science, Buckinghamshire, United Kingdom) with 5 ml of buffer A containing 20 mM imidazole, proteins were applied to the column and the flow-through fraction was collected for analysis. The column was washed with buffer A supplemented with 20 mM imidazole and then with buffer B (8 M Urea, 100 mM sodium phosphate, 10 mM Tris, pH 6.3). Then the column was washed with buffer C (50 mM Tris pH 8.0, 300 mM NaCl, 20 mM imidazole). Finally, the proteins were eluted four times with 1 ml of 200 mM imidazole resolved in buffer C. Protein concentration in the eluted fractions was determined with Bradford assay.

### Statistical Analysis

Data were analyzed using GraphPad Prism 5 (San Diego, CA, USA) Software and were presented as mean ± SEM of at least three independent experiments. The significance of differences was calculated using Students *t*-test and One-way Anova test. *P* value < 0.05 was considered to indicate a significant difference.

## Results

### βSyn Has a Higher Toxicity Threshold Than αSyn When Expressed in Yeast

**Figure 1:**
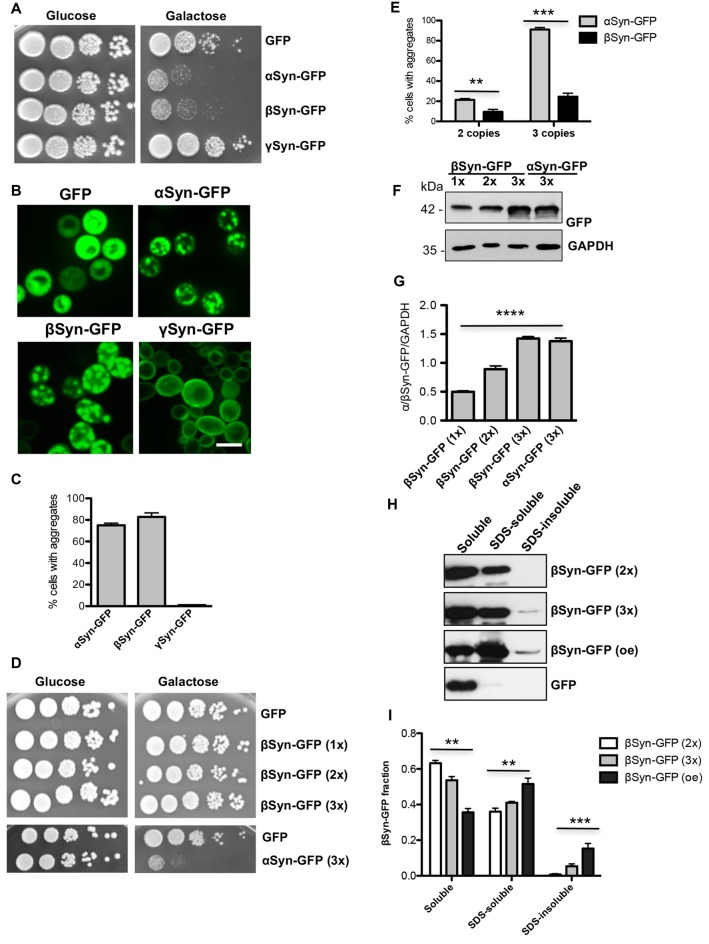
β-synuclein (βSyn)-GFP expression impairs yeast growth. **(A)** Wild type yeast cells (W303) were transformed with a high copy plasmid carrying α-synuclein (αSyn)-GFP, βSyn-GFP and γ-synuclein (γSyn)-GFP under the control of *GAL1*. GFP expressing cells, expressed from the same promoter, served as control. Yeast cells were spotted in 10-fold dilutions on selection plates containing glucose (*GAL1* promoter “OFF”) or galactose (*GAL1* promoter “ON”). **(B)** Live-cell fluorescence microscopy of yeast cells expressing *GAL1*-driven synuclein isoforms from 2μ plasmids. Yeast cells, pre-grown to mid-log phase, were induced in galactose-containing medium and examined for aggregates at 6 h of induction. Scale bar—5 μM. **(C)** Aggregate quantification of yeast cells, expressing synuclein-GFP isoforms. For each strain, the number of cells displaying cytoplasmic foci is presented as percent of the total number of cells. For quantification of aggregation at least 200 cells were counted per strain and per experiment. The results are mean from at least five independent experiments ± SD. **(D)** Spotting assay of yeast cells expressing one (1x), two (2x) or three (3x) copies of βSyn and three copies of αSyn. Yeast cells, expressing GFP were used as a control. The yeast cells were spotted in 10-fold dilutions on selective SC-Ura plates containing 2% glucose and 2% galactose. **(E)** Aggregate quantification of yeast cells, expressing two or three copies of genes, encoding αSyn-GFP and βSyn-GFP at 6 h of induction. Expression of βSyn-GFP from a single copy gene did not result in aggregate formation. Significance of differences was calculated with *t*-test (**p < 0.01; ***p < 0.001; *n* = 3). **(F)** Western blot analysis of crude protein extracts of GFP-tagged proteins, expressed from one, two or three copies of βSyn and three copies of αSyn encoding genes, respectively. GAPDH was used as a loading control. **(G)** Densitometric analysis of the immunodetection of βSyn-GFP relative to the GAPDH loading control. Significance of differences was calculated with One-way Anova test (****p < 0.0001; *n* = 3). **(H)** Distribution of βSyn-GFP levels across different solubility fractions. Yeast cells, expressing βSyn-GFP from two (2x) or three (3x) genomic copies or overexpressing (oe) the protein from high copy plasmid were induced for 6 h in galactose medium. Starting from equal number of cells, crude protein extracts were prepared and fractionated by ultracentrifugation to produce soluble, SDS-soluble and SDS-insoluble fractions. Fractionation of GFP was performed as a control. Equal amounts from all fractions were analyzed by immunoblotting with βSyn or GFP antibody. **(I)** Densitometric analysis of the immunodetection of βSyn-GFP. The relative ratio of each βSyn fraction is normalized to the sum of the three fractions. Significance of differences was calculated with One-way Anova test (**p < 0.01; ***p < 0.001; *n* = 4).

We evaluated the impact of αSyn, βSyn and γSyn GFP-tagged proteins on growth of *Saccharomyces cerevisiae* cells. The expression was controlled by the *GAL1* promoter, which can be repressed in the presence of glucose and induced in the presence of galactose. High level expression from 2μ plasmid of αSyn resulted in the described growth impairment (Outeiro and Lindquist, 2003; Petroi et al., 2012). Cells expressing βSyn were also inhibited in growth, whereas expression of γSyn resulted in wild type yeast growth similar of GFP control (Tenreiro et al., 2016; Figure 1A). Fluorescent microscopy was used to assess, whether the synuclein-induced growth inhibition is accompanied with aggregate formation. αSyn and βSyn expression resulted in formation of similar cytoplasmic inclusions (Figure 1B). Quantification of the cells displaying inclusions revealed similar percentages of αSyn and βSyn expressing cells with inclusions 6 h after induction of protein expression, whereas γSyn expressing cells did not exhibit formation of aggregates (Figure 1C).

Previous studies revealed that a threshold level for αSyn-induced toxicity was reached if expression was driven by three genomic copies of the αSyn encoding gene (Petroi et al., 2012). We aimed to characterize the threshold for βSyn-induced toxicity. Strains were constructed with single, double or triple integrations of βSyn-encoding genes at the single *ura3* locus. Growth assays revealed no growth inhibition by expression of βSyn from single, double or triple genomic copies. In contrast cells expressing αSyn from three copies were impaired in growth (Figure 1D). This growth phenotype correlated with increased aggregate formation. Expression from one copy of βSyn did not result in aggregate formation. Two copies of αSyn and βSyn had lower percentages of cells with aggregates, whereas expression from three copies of αSyn gene resulted in 91% of cells displaying aggregates in comparison with 24% of cells expressing βSyn (Figure 1E). The measured differences in toxicity were not due to different protein levels of the two variants, as observed with Western hybridization after 6 h induction of expression (Figures 1F,G).

The solubility of βSyn in strains differing in the potential for aggregate formation was analyzed. Cells, expressing βSyn from two or three genomic copies and cells, overexpressing βSyn from 2μ plasmid were used. Crude protein extracts were prepared from equal number of cells 6 h after induction of protein expression. The protein extracts were subjected to fractionation to produce soluble, SDS-soluble and SDS-insoluble fractions (Figures 1H,I). Comparison of the different βSyn fractions revealed significant increase towards SDS-insoluble fraction in cells, displaying higher aggregate formation. The results illustrate a direct correlation between βSyn aggregation and the accumulation of insoluble protein species, suggesting that the observed fluorescent foci are indicative for βSyn aggregate formation.

These results demonstrate that expression from three copies of βSyn encoding gene is below the toxicity threshold. βSyn is less toxic with a higher threshold for cytotoxicity and aggregate formation than αSyn.

### βSyn Is Sumoylated in Yeast at Three Lysine Residues Within SUMO Consensus Motifs

**Figure 2:**
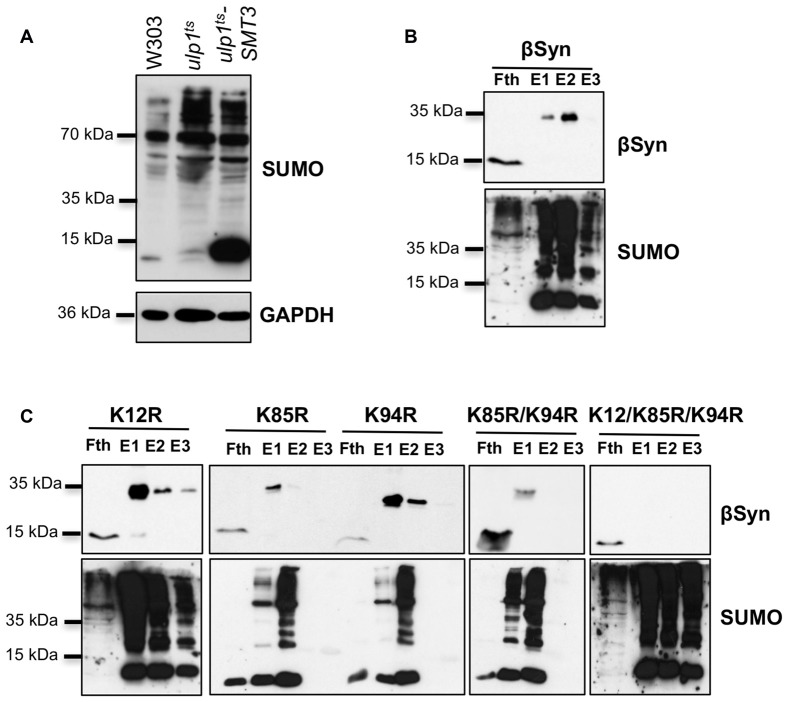
βSyn is sumoylated in yeast.** (A)** Enrichment of sumoylated conjugates in *ulp1^ts^* strain, expressing *SMT3-HIS6*. Western blot analysis of the total protein extract from the *ulp1^ts^* strain, deficient in SUMO-deconjugation, expressing His-tagged *SMT3*. *ulp1^ts^* and W303 were used as controls. SUMO-conjugated proteins were detected using specific SUMO antibody. GAPDH served as a loading control. **(B)** Ni^2+^ pull-down of *ulp1^ts^-SMT3-His6* strain expressing *GAL1*-driven βSyn. Fth: flow-through; E1: Elution 1; E2: Elution 2; E3: Elution 3. The same membrane was stripped and probed with SUMO antibody verifying the Ni^2+^ pull-down. Modified βSyn migrates at ~35 kDa. Unmodified βSyn migrates at ~15 kDa. **(C)** Ni^2+^ pull-down of *ulp1^ts^-SMT3-His6* strain expressing βSyn sumoylation deficient mutants K12R, K85R, K94R, K85R/K94R and K12R/K85/K94R.

αSyn was shown to be efficiently sumoylated in human cells lines and the major sumoylation sites are conserved from yeast to human (Krumova et al., 2011; Shahpasandzadeh et al., 2014). We examined, whether βSyn is sumoylated in yeast cells. *In silico* analysis with SUMOplot analysis predicted three putative SUMO consensus motifs at lysine residues 12, 85 or 94 (K12, K85, K94; Table 3). A temperature-sensitive strain deficient for the gene, encoding the SUMO-deconjugation isopeptidase (*ulp1^ts^*) that expressed His_6_-tagged yeast SUMO protein Smt3 (Petroi et al., 2012) was used for enrichment of SUMO-conjugates (Figure 2A). βSyn was expressed in the *ulp1^ts^-SMT3-His6* strain and SUMO-conjugates were enriched by Ni^2+^-NTA affinity chromatography under denaturing conditions. Immunoblotting analysis with βSyn antibody revealed SUMO-modified βSyn protein with an apparent molecular mass of ~35 kDa (Figure 2B) that could be separated from the non-modified 15 kDa βSyn protein.

**Table 3:** β-synuclein (βSyn) putative sumoylation sites.

Position	Group	Score
K86	AATGL **VKRE** EFPTD	0.93
K95	EFPTD **LKPE** EVAQE	0.91
K12	KGLSM **AKEG** VVAAA	0.62

*The score represents the probability for the SUMO consensus sequence (bold) to be involved in SUMO conjugation. The lysine acceptor site is underlined*.

In order to map the sumoylation sites of βSyn, mutants with single (K12R; K85R; K94R), double (K85/K94R) and triple (K12/K85/K94R) lysine substitutions of the three putative βSyn sumoylation sites were generated. The βSyn lysine mutants were expressed in *ulp1^ts^-SMT3-His6* strain and SUMO-conjugates were purified by Ni^2+^-NTA pull-down as above. Single substitutions of each codon for putative sumoylation site, as well as double substitutions (K85/K94R) did not result in loss of the 35 kDa band. Only when all three putative SUMO-acceptor sites were mutated simultaneously (K12/K85/K94R), complete abolishment of sumoylation was observed (Figure 2C). These results support that βSyn sumoylation *in vivo* comprises all three K12, K85 and K94 lysine residues, which are embedded in a SUMO consensus motif.

### Loss of Sumoylation of βSyn Reduces Inclusion Formation Without Reducing Cytotoxicity

**Figure 3:**
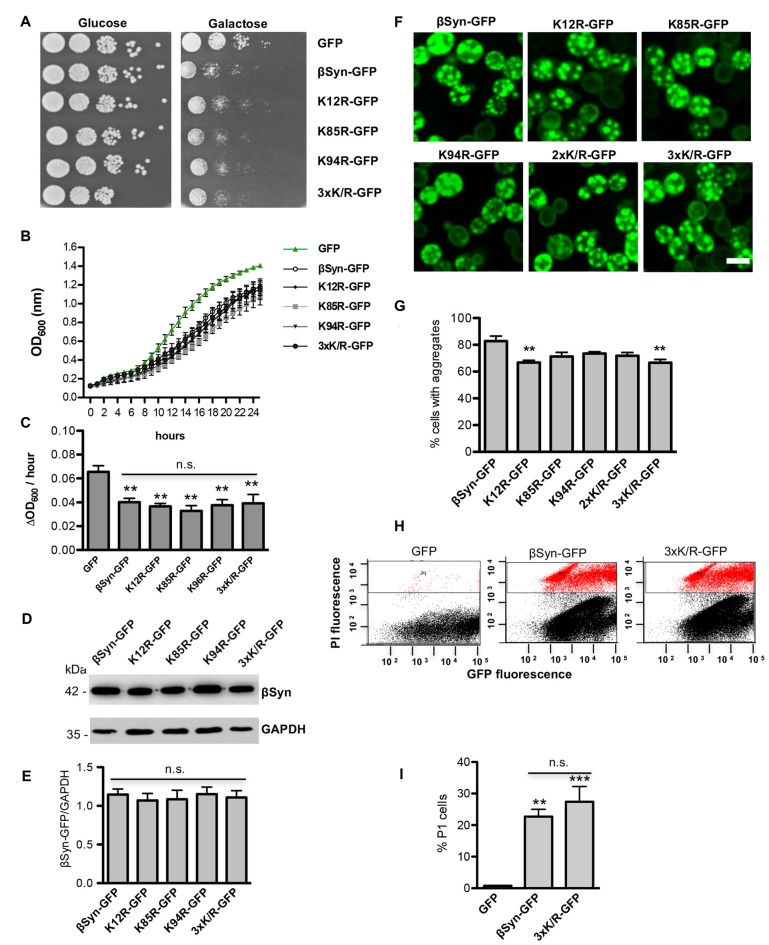
High-copy expression of βSyn-GFP variants in yeast. **(A)** Spotting assay of yeast cells expressing *GAL1*-driven GFP-tagged βSyn and βSyn SUMOylation deficient mutants K12R, K85R, K94R, K85R/K94R (2xK/R) or K12R/K85R/K94R (3xK/R) from 2μ plasmids. GFP expressing plasmid was used as a control. Yeast cells were spotted in 10-fold dilutions on selective plates containing 2% glucose (*GAL1* “OFF”) or 2% galactose (*GAL1* “ON”). **(B)** Growth analysis of yeast cells from **(A)** in galactose-containing medium for 24 h. **(C)** Growth rate of yeast cells from **(B)** in logarithmic phase. Significance of differences to the GFP control was calculated with *t*-test (**p < 0.01; *n* = 4). One-way Anova test revealed no significant differences among the βSyn variants. **(D)** Western blot analysis of protein crude extracts from **(A)** after 6 h induction in galactose-containing medium. GAPDH served as a loading control. **(E)** Densitometric analysis of the immunodetection of βSyn-GFP relative to the GAPDH loading control. One-way Anova test revealed no significant differences among the βSyn variants (*n* = 3). **(F)** Live-cell fluorescence microscopy of yeast cells expressing *GAL1*-driven βSyn-GFP variants from 2μ plasmids. Yeast cells, pre-grown to mid-log phase, were induced in galactose-containing medium and examined for aggregates at 6 h of induction. Scale bar – 5 μM. **(G)** Aggregate quantification of yeast cells, expressing βSyn-GFP variants. For quantification of aggregation at least 200 cells were counted per strain and per experiment. The results are mean from at least four independent experiments ± SD. Significance of differences was calculated with *t*-test (**p < 0.01 vs. βSyn). **(H)** Propidium iodide (PI) fluorescence intensity of cells expressing βSyn, 3xK/R and GFP (control) after 20 h induction of expression, assessed by flow cytometry analysis. **(I)** Quantification of PI-positive yeast cells with higher fluorescent intensities (P1) than the background is presented. Significance of differences to the GFP control was calculated with *t*-test (**p < 0.01; ***p < 0.001; *n* = 4).

We examined whether modification of βSyn as a direct SUMO target affects βSyn-induced growth inhibition in yeast. Wild type βSyn, the single lysine mutants (K12R; K85R; K94R), as well as the triple lysine mutant (K12/K85/K94R) were expressed in yeast. The effect of abolishing the sumoylation acceptor sites of βSyn on yeast growth was examined with spotting test (Figure 3A). Yeast cells were inhibited in growth and no significant differences were observed, when single SUMO acceptor sites or all three SUMO acceptor sites were substituted. Growth in liquid medium resulted in similar effects (Figures 3B,C). Cells, expressing βSyn or sumoylation deficient mutants revealed reduced growth in comparison to the GFP control, however no significant differences in growth were observed among the βSyn variants. Immunoblotting analysis revealed that all proteins were expressed at similar levels after 6 h induction of expression (Figures 3D,E). Fluorescent microscopy studies were performed to assess the aggregate formation of different βSyn variants. Quantification of the number of cells with inclusions revealed a significant reduction in cells displaying K12R, as well as K12/K85/K94R mutants (Figures 3F,G). In order to assess, whether the differences in aggregate formation correlate with changes in cytotoxicity, propidium iodide (PI) staining for membrane permeability was performed as a sensitive method for quantification of yeast viability. Flow cytometry measurements of cells, expressing βSyn and K12/K85/K94R, showed a significantly increased number of PI-positive cells in comparison to the GFP control (Figures 3H,I). No significant differences between cells expressing βSyn and the triple lysine mutant were observed. These data correlate with the results from the growth assays and reveal that SUMO modifications of βSyn enhances aggregate formation of the protein without significantly affecting cell growth and viability. This suggests that βSyn aggregate formation and cytotoxicity are part of different molecular mechanisms.

### Sumoylation Protects Against βSyn-Induced Toxicity

**Figure 4:**
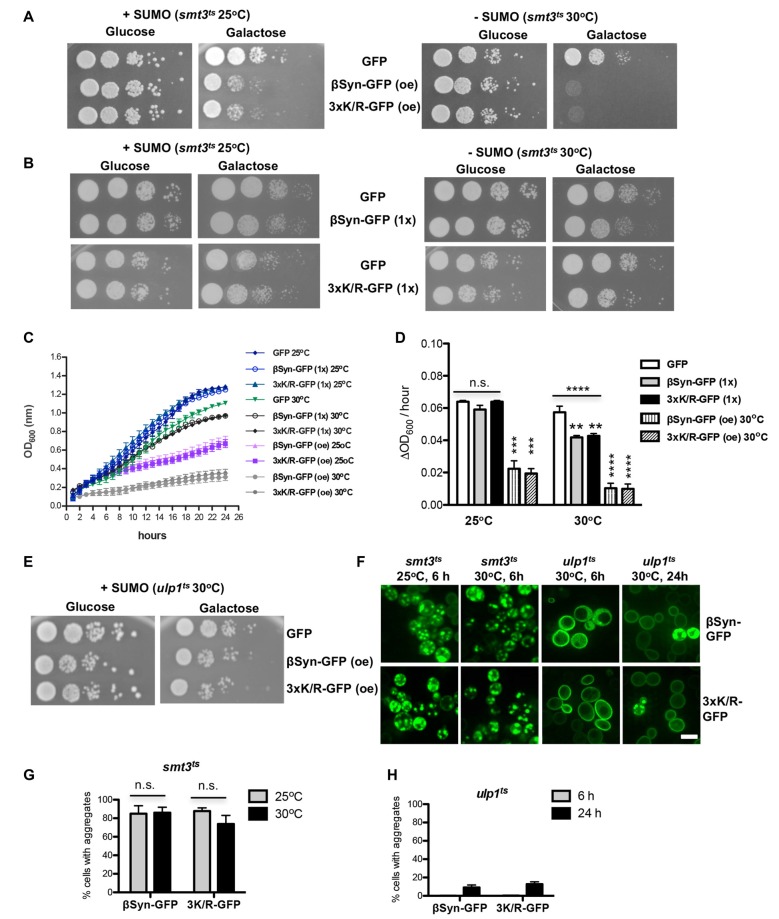
Impairment of sumoylation increases βSyn-GFP induced toxicity. **(A)** Spotting assay of *smt*3^ts^ mutant strain expressing βSyn-GFP and 3xK/R-GFP at permissive (25°C; +SUMO) or restrictive temperature (30°C; −SUMO). *GAL1*-driven synucleins are expressed from 2μ plasmid (oe-overexpression). GFP, expressed from the same promoter, is used as a control. Yeast cells were spotted in 10-fold dilutions on selection plates containing glucose (*GAL1* promoter “OFF”) or galactose (*GAL1* promoter “ON”). **(B)** Spotting assay of *smt*3^ts^ mutant strain expressing one copy (1x) of βSyn-GFP or 3xK/R-GFP. Yeast cells, expressing GFP were used as a control. **(C)** Growth analysis of yeast cells from **(A,B)** in galactose-containing medium for 24 h at the indicated temperatures. **(D)** Growth rate of yeast cells in logarithmic phase. Significance of differences was calculated with One-way Anova test or *t*-test, relative to the GFP control (**p < 0.01; ***p < 0.001; ****p < 0.0001; *n* = 4). **(E)** Spotting assay of *ulp1^ts^* mutant strain expressing βSyn-GFP and 3xK/R-GFP from 2μ plasmid at 30°C. **(F)** Live-cell fluorescence microscopy of yeast cells expressing *GAL1*-driven synuclein isoforms from 2μ plasmids in *smt*3^ts^ strain and *ulp1^ts^* strain. Yeast cells, pre-grown to mid-log phase, were induced in galactose-containing medium and examined for aggregates at indicated temperatures and hours after induction. Scale bar—5 μM. **(G)** Aggregate quantification of yeast cells, expressing synuclein-GFP isoforms in *smt*3^ts^ strain at 25°C and 30°C. For each strain, the number of cells displaying cytoplasmic foci is presented as percent of the total number of cells. The results are mean from at least four independent experiments ± SD. **(H)** Aggregate quantification of yeast cells, expressing synuclein-GFP isoforms in *ulp1^ts^* strain 6 h and 24 h after induction of protein expression.

The finding that SUMO modification at βSyn acceptor sites did not change cytotoxicity suggested that sumoylation of additional target proteins or cellular pathways might be involved in cytoprotection against βSyn-induced toxicity. We assessed the effect of sumoylation on βSyn-induced toxicity using a yeast strain with a temperature sensitive SUMO-encoding gene (*smt3^ts^*). Growth of yeast *smt3^ts^* cells at restrictive temperature (30°C) results in accumulation of non-functional SUMO-conjugates (Dieckhoff et al., 2004; Shahpasandzadeh et al., 2014). A growth assay was performed with cells, expressing βSyn or K12/K85/K94R mutant from high copy plasmid at permissive temperature (25°C) and restrictive temperature (30°C; Figure 4A). Impairment of sumoylation at 30°C only slightly affected growth of the control strain expressing GFP, whereas expression of βSyn or K12/K85/K94R mutants resulted in drastic growth inhibition under these conditions. Importantly, at permissive temperature cells that were not depleted of functional SUMO were less affected by expression of βSyn and derivatives. Mutant *smt3^ts^* yeast strains with single integration of βSyn or 3xK/R-encoding genes at the *ura3* locus were constructed. Expression from single copy of βSyn or K12/K85/K94R-encoding gene is lower than the toxicity threshold (Figure 1D). Accordingly, growth assays revealed no retardation at 25°C (Figure 4B). Inhibition of sumoylation at 30°C slightly affected growth of the strains expressing βSyn or K12/K85/K94R. Quantitative growth assays in liquid cultures were performed with the *smt3^ts^* yeast cells, expressing βSyn or K12/K85/K94R from single gene copy (Figures 4C,D). Analysis of the growth rate revealed significant differences in growth compared to the GFP control when sumoylation is impaired. These results indicated gain of βSyn toxicity in strains expressing non-toxic levels of βSyn or K12/K85/K94R under conditions of impaired sumoylation. This suggests a protective role of sumoylation for βSyn-induced toxicity that is not dependent on direct modification of the protein.

Next, we examined whether increased sumoylation affects βSyn-induced cytotoxicity. We used the *ulp1^ts^* strain, defective for SUMO de-conjugation, to enrich the pool of sumoylated proteins. Expression of βSyn or K12/K85/K94R resulted in significantly reduced growth inhibition (Figure 4E). This result revealed that up-regulation of sumoylation partially rescues the growth defect of yeast cells, expressing βSyn.

We tested, whether there is a correlation between growth inhibition and aggregate formation of βSyn and K12/K85/K94R variant. Aggregate formation was followed by life-cell microscopy and the number of cells with aggregates was counted (Figure 4F). Expression of βSyn variants in *smt3^ts^* cells in presence or absence of functional SUMO did not result in changes of the percentage of cells with aggregates 6 h after induction (Figure 4G). However, expression of βSyn and K12/K85/K94R in *ulp1^ts^* cells resulted in complete loss of aggregation after 6 h of induction and increased cytoplasmic staining (Figures 4F,H). Formation of aggregates was observed as late as 24 h after protein induction and there was a strong decrease of the percentage of cells with aggregates in comparison with *smt3^ts^* background (Figure 4G) or wild type W303 background (Figure 1C).

Our findings support a complex interplay between sumoylation, βSyn-induced cytotoxicity and aggregate formation. Downregulation of functional cellular SUMO pools strongly inhibits yeast growth without affecting efficient aggregate formation. In contrast, higher levels of sumoylated proteins by inhibition of SUMO de-conjugation enzymes prevented aggregate formation but still caused significant cellular growth inhibition. These effects are not dependent on direct sumoylation of βSyn, since the growth and aggregation propensity of βSyn and K12/K85/K94R-expressing cells is influenced similarly. The results suggest an indirect effect of SUMO on βSyn-induced cytotoxicity by modification of other SUMO-target proteins that affect the aggregation or cytotoxicity of βSyn.

### βSyn Aggregates Are Cleared Mainly By the 26S Proteasome

Both autophagy and UPS are implicated in degradation of αSyn and manifestation of PD. Inefficient protein clearance may lead to accumulation of toxic protein species and is sufficient to trigger neurotoxicity (Xilouri et al., 2013). In yeast, autophagy represents the major pathway for degradation of αSyn aggregates (Petroi et al., 2012; Tenreiro et al., 2014) and this process is promoted by sumoylation (Shahpasandzadeh et al., 2014).

**Figure 5:**
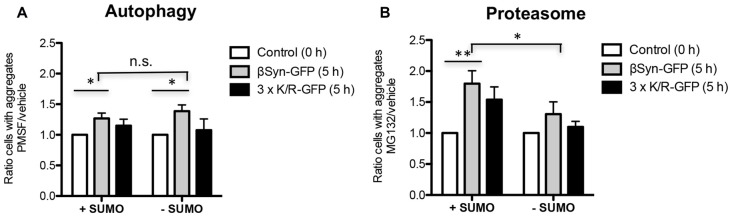
βSyn aggregate clearance after promoter shut-off. Quantification of cells displaying aggregates of βSyn-GFP or 3xK/R-GFP upon inhibition of autophagy by phenylmethanesulfonyl fluoride (PMSF) **(A)** or proteasome by MG132 **(B)**. Yeast cells were incubated in 2% galactose-containing media for 4 h either at permissive (25°C; +SUMO) or restrictive temperature (30°C; −SUMO) and shifted to 2% glucose-containing media supplemented with 1 mM PMSF dissolved in EtOH **(A)** or 75 μM MG132, dissolved in dimethyl sulfoxide (DMSO) **(B)**. Control cells were supplemented with vehicles only (EtOH or DMSO). Cells with aggregates were counted after 5 h *GAL1*-promoter shut-off and presented as a ratio to the control. Significance of differences was calculated with *t*-test (*p < 0.05; **p < 0.01; *n* = 4).

We assessed the mechanism of βSyn aggregate clearance mediated by the autophagy/vacuolar and the ubiquitin-proteasome pathways. The impact of blocking these systems by drug treatments was studied. Expression of βSyn and K12/K85/K94R in *smt3^ts^* cells was induced for 4 h at permissive (25°C; +SUMO) or restrictive temperature (30°C; −SUMO). Promoter shut-off was achieved by shifting the cells to glucose-containing medium that represses the *GAL1* promoter. PMSF was used as an inhibitor of autophagy/vacuolar pathway as described previously (Petroi et al., 2012; Kleinknecht et al., 2016; Figure 5A). Cells were imaged 5 h after promoter shut-off. Inhibition of autophagy resulted in increased number of cells with βSyn or K12/K85/K94R aggregates relative to the control. This suggests that the autophagy/vacuolar pathway contribute to βSyn aggregate clearance. Inhibition of sumoylation resulted in similar aggregate clearance.

The contribution of the UPS to βSyn degradation was analyzed by inhibiting this system with the proteasome inhibitor MG132 (Figure 5B). In the presence of functional SUMO, there was almost two-fold increase of cells with βSyn aggregates in comparison to the control, when the proteasome is not inhibited. These data suggest a major contribution of the UPS to βSyn or K12/K85/K94R aggregate clearance. In contrast to autophagy, downregulation of sumoylation had a significant impact on the aggregate clearance by the proteasome, diminishing the contribution of this system on βSyn aggregate clearance.

These results suggest that βSyn aggregates in yeast are cleared mainly by the proteasome with a smaller contribution of autophagy/vacuolar pathway. The proteasomal clearance of βSyn aggregates is not significantly affected by direct sumoylation of βSyn lysine residues. Downregulation of sumoylation significantly reduces βSyn aggregate clearance by the proteasome, however, does not affect the autophagy/vacuolar mediated aggregate clearance.

### Sumoylation Affects βSyn Protein Turnover

**Figure 6:**
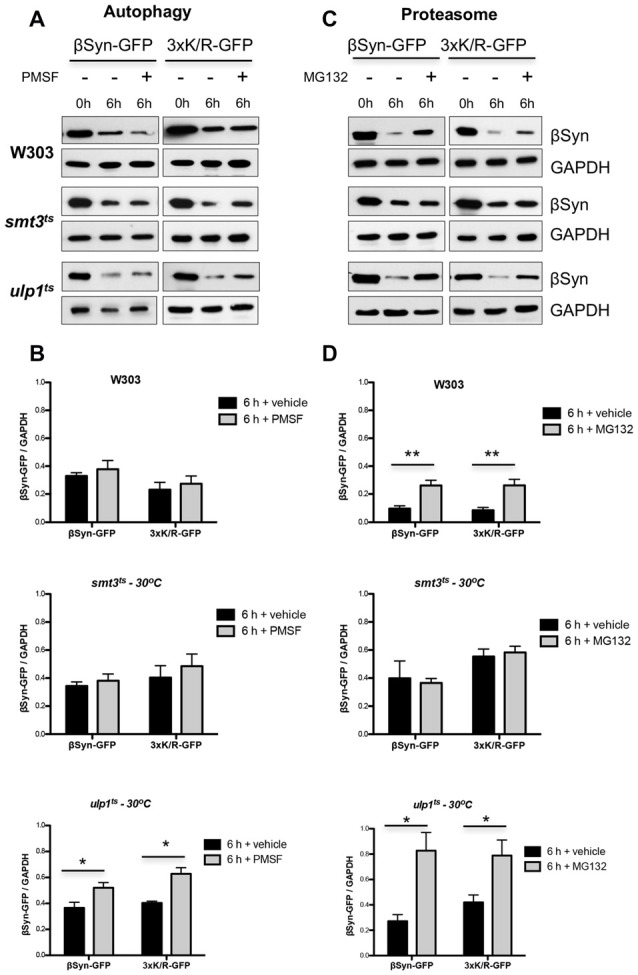
Effect of sumoylation on βSyn protein stability. Yeast cells, expressing βSyn-GFP or 3xK/R-GFP variant in the indicated strains were induced for 4 h in galactose medium (*GAL1* “ON”) and then transferred to glucose containing medium (*GAL1* “OFF”). The glucose medium was supplemented with 1 mM PMSF **(A)** or 75 μM MG132** (C)**. Immunoblotting analysis was performed at the indicated time points after promoter shut-off with βSyn antibody and GAPDH as a loading control. A representative result is shown from at least three independent experiments. **(B,D)** Densitometric analysis of the immunodetection of βSyn relative to the GAPDH loading control. Significance of differences was calculated with *t*-test (*p < 0.05; **p < 0.01; *n* = 3).

We next analyzed the effect of sumoylation on the protein stability of βSyn and K12/K85/K94R by promoter shut-off experiments. The contribution of the autophagy/vacuole and the proteasome pathway was examined as described above by inhibiting each system with drug treatment. Protein expression was induced for 4 h in galactose medium, then the cells were shifted to glucose medium (*GAL1*-“OFF”) and protein crude extracts were prepared 6 h after promoter shut-off. Assays were performed in W303 wild type, *smt3^ts^* and *ulp1^ts^* strains at restrictive temperature (30°C). Immunoblotting analysis was performed and steady-state levels of βSyn variants were quantified (Figure 6). Inhibition of autophagy/vacuolar pathway by PMSF did not result in changes of βSyn or K12/K85/K94R protein levels in wild type W303 background in comparison to the control (Figures 6A,B). Impairment of functional SUMO (*smt3^ts^*) did not significantly affect the protein levels of both βSyn variants, when autophagy was inhibited. However, inhibition of the vacuolar/autophagy pathway resulted in significant increases of the protein levels of both βSyn variants, when the pool of sumoylated proteins was up-regulated in *ulp1^ts^* strain.

In contrast to autophagy, inhibition of the proteasome with MG132 in presence of functional SUMO in yeast wild type background had a strong impact on the protein stability (Figures 6C,D). Increased protein levels of both βSyn variants were detected in comparison to the control, suggesting involvement of the UPS in degradation of βSyn and K12/K85/K94R proteins. Importantly, impairment of functional SUMO in *smt3^ts^* strain did not alter ßSyn protein levels 6 h after promoter shut-off, when the proteasome was impaired, indicating that inhibition of sumoylation suppressed the proteasomal degradation of βSyn and K12/K85/K94R derivative. Proteasome impairment resulted in significantly compromised degradation of the proteins in *ulp1^ts^* strain, when the pool of sumoylated proteins is up-regulated, revealing the major impact of the proteasome and functional sumoylation machinery on βSyn degradation.

These findings suggest that the 26S-proteasome represents the major pathway for degradation of βSyn in the cells. Functional sumoylation machinery is required for the efficient degradation of the protein by the 26S-proteasome, indicating a cross-talk between sumoylation and UPS in βSyn protein homeostasis. This process is not dependent on direct SUMO modification of βSyn at K12, K85 or K94 acceptor sites. Sumoylation promotes the proteasomal degradation of βSyn. Up-regulation of the sumoylation pool promotes in addition the degradation by autophagy/vacuole.

### Downregulation of Sumoylation Increases the Accumulation of Ubiquitinated Cellular Proteins

**Figure 7:**
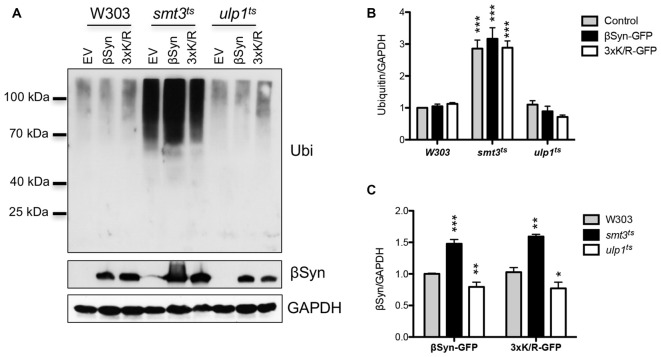
Interplay between sumoylation and ubiquitination. **(A)** Western blot analysis of crude protein extracts from yeast cell, expressing βSyn-GFP and 3xK/R-GFP mutant in W303, *smt3^ts^* and *ulp1^ts^* strains at 30°C, probed with ubiquitin antibody, detecting poly- and mono-ubiquitinated proteins. The same membrane was stripped and probed with βSyn antibody. GAPDH was used as a loading control. **(B)** Densitometric analysis of the immunodetection of ubiquitin or βSyn **(C)**, relative to the GAPDH loading control. Significance of differences was calculated with *t*-test (***p < 0.001; **p < 0.01; *p < 0.05 vs. W303 wild type background; *n* = 3).

Sumoylation is involved in protein homeostasis by its interplay with the UPS (Liebelt and Vertegaal, 2016). It was examined, whether compromised degradation of βSyn by the proteasome is connected to misregulation of UPS when cellular sumoylation is decreased. We followed the accumulation of ubiquitinated substrates in yeast cells in presence and absence of βSyn expression and tested if downregulation of the cellular pool of functional SUMO affects the levels of ubiquitin conjugates. Immunoblotting analysis was performed with protein extracts from yeast cells, expressing βSyn, K12/K85/K94R or empty vector control (Figure 7A). Inhibition of sumoylation in *smt3^ts^* strain revealed accumulation of ubiquitinated proteins, whereas up-regulation of the sumoylation pool by inhibition of the SUMO de-conjugation resulted in similar levels of ubiquitinated proteins as in W303 wild type yeast cells (Figure 7B). Expression of βSyn variants did not affect the accumulation of ubiquitinated proteins in comparison to the empty vector control. Importantly, the steady-state levels of βSyn variants differed between the yeast strains. Expression of βSyn or K12/K85/K94R in *smt3^ts^* cells resulted in significantly increased protein accumulation in comparison to the wild type W303 strain (Figure 7C), whereas up-regulation of the sumoylation in *ulp1^ts^* cells decreased the protein levels. The increased protein levels correlated with accumulation of ubiquitinated substrates in *smt3^ts^* cells.

These findings showed that reduction of available functional SUMO has a strong impact on the steady-state levels of ubiquitin conjugates, including proteasomal degradation. This presumably leads to accumulation of soluble βSyn protein in the cell and supports that sumoylation protects against βSyn-induced toxicity by promoting the degradation of soluble as well as aggregated protein.

## Discussion

The budding yeast *Saccharomyces cerevisiae* was used as a prototypic eukaryotic cell to investigate the mechanism of βSyn turnover and the effect of sumoylation on βSyn-induced cytotoxicity, aggregation and protein stability. Accumulating evidence suggested that besides αSyn, also βSyn is involved in the pathogenesis of aggregopathies such as PD. In the current study, we analyzed βSyn lysine residues required for covalent attachment of SUMO, as well as the effect of the cellular sumoylation pool on βSyn-induced toxicity, aggregate formation and stability.

Initial reports on βSyn in double transgenic mice suggested that βSyn is less prone to aggregation than αSyn and may counteract the αSyn aggregation (Hashimoto et al., 2001). Recent studies reported that βSyn contributes to neuronal degeneration and suggest a possible toxic gain-of-function of βSyn. It was shown that βSyn forms Proteinase K resistant aggregates in primary cultured neurons and dopaminergic neurons in rat brains and to cause neurotoxicity (Taschenberger et al., 2013). Importantly, this effect was dependent on the accumulation of the protein over time and after 8 weeks of βSyn overexpression, similar neurodegenerative effect to that of αSyn was observed. Another study reported on two mutations of βSyn, P123H and V70M, linked to DLB that may stimulate neurodegeneration in transgenic mice by itself or via αSyn aggregation (Fujita et al., 2010). These studies reveal that βSyn may possess gain-of-function pathogenic properties on its own or act synergistically with αSyn and that alteration of βSyn levels in the cell might play a role in a broad range of synucleinopathies.

Budding yeast is an established model system to study the molecular mechanisms of PD and other synucleinopathies (Menezes et al., 2015; Popova et al., 2015). Expression of human αSyn in yeast induces growth inhibition and aggregate formation, similar to the pathology of the disease (Outeiro and Lindquist, 2003; Petroi et al., 2012). Recently, yeast was used to investigate the molecular mechanism of βSyn-induced toxicity (Tenreiro et al., 2016). Expression of βSyn was toxic and resulted in formation of cellular aggregates resembling the αSyn aggregates. βSyn expression induced defects in similar pathways as αSyn, including ER-to-Golgi trafficking defects and increased oxidative stress. Importantly, co-expression of αSyn and βSyn resulted in formation of heterodimers and enhanced toxicity due to additive accumulation of toxic protein species. Co-expression of αSyn and βSyn strongly inhibited yeast vegetative growth, preventing the in-depth analysis of the molecular effects of α/ßSyn co-expression (Tenreiro et al., 2016).

The increase in αSyn-mediated cytotoxicity is dose-dependent resulting in a threshold for toxicity (Outeiro and Lindquist, 2003; Petroi et al., 2012). The thresholds for βSyn and αSyn mediated toxicity differed considerably in yeast. Three copies of the *GAL1* promoter driven αSyn gene integrated into the yeast genome inhibited cellular growth (Petroi et al., 2012), but three copies with the same promoter for βSyn were not sufficient for a significant growth effect. Overexpression of βSyn significantly inhibited yeast growth and induced formation of cellular aggregation, similar to αSyn, corroborating that βSyn has a higher threshold for toxicity and aggregation than αSyn.

PTMs including phosphorylation, ubiquitination, nitration, glycosylation, acetylation or sumoylation play an important role in regulation of αSyn function and stability (Giasson et al., 2000; Shimura et al., 2001; Fujiwara et al., 2002; Hasegawa et al., 2002; Dorval and Fraser, 2006; de Oliveira et al., 2017). Sumoylation can change sub-cellular protein localization, alter protein-protein interactions or change the solubility of the protein. Sumoylation is a dynamic process and the levels of sumoylated proteins are regulated by different stresses (Enserink, 2015). Sumoylation is directly involved in maintaining protein homeostasis and is closely linked to neurodegeneration, exhibiting a protective role against αSyn-induced toxicity and inclusion formation in yeast (Shahpasandzadeh et al., 2014).

**Figure 8:**
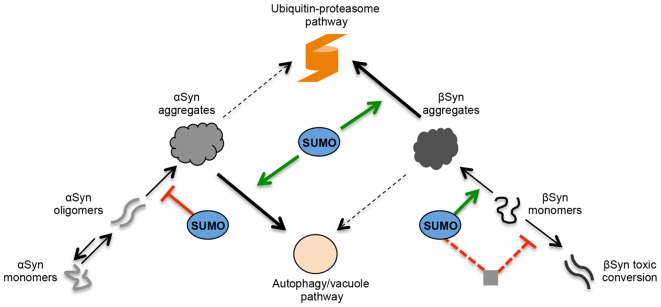
Role of SUMO in the regulation of αSyn and βSyn turnover and toxicity in yeast. Schematic representation of αSyn and βSyn aggregation pathways. Under pathological conditions, αSyn can undergo oligomerization. A dynamic equilibrium exists between monomeric and multimeric forms of αSyn. The oligomeric species represent intermediates of the aggregation process and are suggested to be the most toxic forms of αSyn. SUMO exhibits a protective role against αSyn-induced toxicity. Sumoylation increases the solubility αSyn and decreases the aggregation of the protein. In contrast, βSyn aggregate formation and cytotoxicity are part of different molecular mechanisms. A fraction of accumulated βSyn might gradually undergo structural conversion and form toxic oligomers or fibrils. In contrast to αSyn, these might not be intermediate species in the aggregation process but might be part of an independent molecular pathway. Sumoylation of βSyn promotes the aggregate formation of the protein without affecting the cytotoxicity. The presence of functional cellular sumoylation machinery protects against βSyn toxicity independently of βSyn sumoylation. Sumoylation of additional target proteins or cellular pathways might be involved in the cytoprotection by inhibiting the formation of βSyn toxic species. αSyn and βSyn aggregates are cleared predominantly by two different pathways. The main pathway for αSyn aggregate clearance is autophagy, whereas ubiquitin-proteasome system (UPS) is the major degradation pathway for βSyn aggregates. Autophagy/vacuolar pathway has a minor contribution to βSyn aggregate clearance. Presence of functional SUMO protects against βSyn as well as αSyn toxicity by promoting the degradation of the proteins. Sumoylated αSyn is primarily targeted to the autophagy pathway, whereas presence of functional SUMO promotes the degradation of βSyn monomers and βSyn aggregates by the ubiquitin-proteasome pathway.

Three lysine residues within consensus SUMO motifs are used for PTM of βSyn in yeast. Inhibition of sumoylation at the single K12 site as well as at all three SUMO sites resulted in decreased aggregate formation, suggesting that sumoylation promotes aggregation. The results are based on mutagenesis analysis changing the codons for the consensus sumoylation sites of βSyn. Cell growth or viability was not affected when sumoylation was inhibited. This supports that βSyn mediated aggregate formation and cytotoxicity represent unlinked molecular functions. Global inhibition of the cellular sumoylation resulted in significant increase in βSyn-induced toxicity without changes in aggregate formation. Functional sumoylation therefore protects yeast cells against βSyn-induced toxicity without affecting the aggregate propensity of the protein (Figure 8). In line with our observations, expression of V70M and P123H βSyn mutants, which are associated with sporadic and familial cases of DLB, increased the aggregate formation without enhancing the toxicity in yeast (Tenreiro et al., 2016). Accumulated βSyn may undergo additional PTMs such as phosphorylation or acetylation and gain pathogenic properties. A fraction of βSyn might adopt a different structure and form toxic oligomers or fibrils. In contrast to αSyn this might not necessarily lead to aggregates. The cellular environment might be relevant, because it is known that βSyn can form fibrils at mild acidic pH as a result of pH dysregulation due to oxidative stress or in acidic organelles such as endosomes or lysosomes/vacuoles (Moriarty et al., 2017). Alteration of βSyn may enhance synergistically the toxicity of αSyn and promote PD pathology. Another possible explanation is that the disintegration between toxicity and aggregation is due to the co-existence of toxic aggregates and non-toxic “protective” aggregates. Sumoylation might protect against cytotoxicity by promoting the conversion of toxic into protective aggregates.

The protective role of sumoylation against αSyn-induced toxicity is significantly different from βSyn. Sumoylation increases the solubility αSyn and thereby decreases the aggregation of the protein (Krumova et al., 2011). In contrast to βSyn, direct modification of αSyn by SUMO at the conserved modification sites K96 and K102 reduced the formation of inclusions and simultaneously protected against cytotoxicity. Therefore, sumoylation promotes βSyn but reduces αSyn aggregation. In contrast, the cellular sumoylation machinery is equally important for protection against βSyn as well as αSyn, because inhibition of cellular sumoylation had a similar negative impact on growth of yeast cells expressing either βSyn or αSyn (Shahpasandzadeh et al., 2014).

One of the major factors that lead to PD pathogenesis is decreased degradation of αSyn protein by one of the two major proteolytic systems—the autophagy/lysosome system or UPS (Xilouri et al., 2013; Vilchez et al., 2014). This contributes to increased protein levels and aggregation. Yeast has been extensively used for examination of αSyn degradation due to the high conservation of functions and pathways, involved in protein quality control between yeast and higher eukaryotes, including humans. Autophagy is the major pathway for αSyn aggregate clearance in yeast (Petroi et al., 2012; Tenreiro et al., 2014). Sumoylation of αSyn promoted aggregate clearance by autophagy, whereas phosphorylation at S129 triggered increased ubiquitination and degradation of the protein by the UPS (Shahpasandzadeh et al., 2014). Here, we demonstrated that UPS is the major degradation pathway, responsible for clearance of βSyn aggregates. Sumoylation promoted the aggregate clearance by the UPS significantly, whereas autophagy/vacuolar pathway had a minor contribution to βSyn aggregate clearance that was not dependent on a functional sumoylation pool (Figure 8). These results indicate that αSyn and βSyn aggregates have different mechanisms for aggregate clearance.

The effect of SUMO on βSyn protein stability is consistent with the aggregate clearance assays. Inhibition of the proteasome significantly increased the level of βSyn. Downregulation of sumoylation stabilized the βSyn protein and abolished its degradation through the UPS, whereas upregulation of the sumoylation had an opposite effect on protein stability. Similarly, sumoylation promotes the degradation of soluble αSyn monomers in yeast that occurs through both degradation pathways (Shahpasandzadeh et al., 2014). These findings highlight the complex regulative role of SUMO in balancing the protein levels of αSyn and βSyn.

Autophagy and the ubiquitin-dependent degradation pathways do not act independently from each other. In the process of selective autophagy, ubiquitination can target proteins not only to the 26S-proteasome but also for autophagic degradation (Kirkin et al., 2009). Inactive 26S-proteasomes can be degraded by the autophagy in a newly discovered pathway termed “proteaphagy” (autophagy of proteasomes), initially described in plants and yeast (Marshall et al., 2015, 2016) and recently reported in mammalian cells (Cohen-Kaplan et al., 2016). Inactivation of UPS is one of the major causes for PD pathology. A proposed mechanism for UPS dysfunction is the direct binding of soluble oligomeric or aggregated species of αSyn with the 26S-proteasome that inhibit its activity (Xilouri et al., 2013). Thus, a possible scenario for αSyn clearance is degradation of the protein together with the inactive proteasomes via the proteaphagy pathway. βSyn has no direct effect on the 26S ubiquitin-dependent degradation and does not bind to the 26S proteasome (Snyder et al., 2005). Thus, higher protein loads of βSyn can be processed by the 26S-proteasome. This might account for the different involvement of the two degradation pathways in the clearance of αSyn and βSyn.

An important contribution of SUMO to protein homeostasis is its interplay with the UPS (Liebelt and Vertegaal, 2016). SUMO-acceptor lysine residues can be targets for ubiquitin conjugation. Thus, sumoylation can directly antagonize the degradation by UPS by a competition with ubiquitin at the same lysine residues, as first shown for IκB-α (Desterro et al., 1998), or can function cooperatively with ubiquitination in a sequential cycle that regulates the function, localization and stability of the protein, as reported for PCNA (Hoege et al., 2002). SUMO can also function as a recognition signal for SUMO-targeted ubiquitin ligases, thus mediating ubiquitin-dependent degradation by the proteasome (Uzunova et al., 2007; Tatham et al., 2008). Downregulation of sumoylation in our studies resulted in significant accumulation of ubiquitinated conjugates and increased levels of βSyn protein in the cell. Expression of βSyn did not affect the steady-state levels of ubiquitin conjugates, consistent with previous findings (Snyder et al., 2005). This indicates that elevated levels of soluble βSyn may account for the increased βSyn-induced toxicity and sumoylation has a protective role by promoting the degradation of the protein.

The complex functional interaction of SUMO and the UPS may also contribute to off-target effects of sumoylation on βSyn protein stability and toxicity. Sumoylation might regulate protein stability or function of additional target proteins and thus indirectly affect the cytotoxicity of βSyn (Figure 8). The off-target effect of SUMO may co-exist with the direct effect of sumoylation on βSyn toxicity. It should be noted that the analysis of temperature sensitive mutant strains can evoke additional indirect effects on βSyn protein quality control.

In this study, we provide evidence that the molecular basis of βSyn-induced cytotoxicity and accumulation in the cell is not directly linked to βSyn aggregate formation. Aggregate formation and cytotoxicity are both linked to sumoylation. Sumoylation determines the accumulation of βSyn and therefore its cytotoxicity. Our data strongly support that the major molecular pathway involved in βSyn turnover and clearance of soluble as well as aggregated proteins is the 26S proteasome.

## Author Contributions

BP and GHB designed the research. BP, AK, PA, JM and DW performed and analyzed the experiments. BP and GHB wrote the manuscript, with contributions from AK.

## Conflict of Interest Statement

The authors declare that the research was conducted in the absence of any commercial or financial relationships that could be construed as a potential conflict of interest.
